# Review of emerging imaging findings to reveal a broader spectrum of lesions in AMD

**DOI:** 10.1007/s00417-026-07335-9

**Published:** 2026-06-25

**Authors:** Enrico Borrelli, Giovanni Neri, Alessandro Berni, Chiara Olivieri, Jay Chhablani, Giulia Corradetti, Rosa Dolz-Marco, Gregor S. Reiter, Dinah Zur, Francesco Bandello, Michele Reibaldi

**Affiliations:** 1 Department of Surgical Sciences, University of Turin, Turin, Italy; 2 Department of Ophthalmology, “City of Health and Science” Hospital, Turin, Italy; 3 Department of Ophthalmology, Faculty of Medicine, Biruni University, İstanbul, Turkey; 4 IRCCS San Raffaele Scientific Institute, Milan, Italy; 5 Vita-Salute San Raffaele University, Milan, Italy; 6 Department of Ophthalmology, University of Pittsburgh School of Medicine, Pittsburgh, PA USA; 7 Department of Ophthalmology, David Geffen School of Medicine at UCLA, Los Angeles, CA USA; 8 Doheny Eye Institute, Pasadena, CA USA; 9 Unit of Macula, Oftalvist Clinic, Valencia, 46004 Spain; 10 Department of Ophthalmology and Optometry, Medical University of Vienna, Vienna, Austria; 11 Department of Ophthalmology, Mayo Clinic, Rochester, MN USA; 12 Faculty of Medical and Health Sciences, Tel Aviv University, Tel Aviv, Israel; 13 Division of Ophthalmology, Tel Aviv Sourasky Medical Center, Tel Aviv, Israel; 14 Department of Ophthalmology, University of Turin, Via Cherasco, 23, Turin, Italy

**Keywords:** Age-related macular degeneration, Classification, Imaging biomarkers, Intermediate AMD, Geographic atrophy, Macular neovascularization

## Abstract

Age-related macular degeneration (AMD) is the leading cause of irreversible vision loss in developed countries, with prevalence expected to reach nearly 288 million by 2040. Accurate classification of AMD is critical for patient care and clinical research, guiding prognosis, therapeutic strategies, and the design of clinical trials. A widely adopted framework, the Beckman classification, stratifies AMD based primarily on color fundus photography (CFP) findings, defining stages from early to late disease. While simple and clinically applicable, such systems do not account for several key phenotypes revealed by advances in multimodal imaging. Such novel phenotypes include reticular pseudodrusen (RPD), acquired vitelliform lesions (AVL), non-exudative macular neovascularization (MNV), incomplete retinal pigment epithelium and outer retina atrophy (iRORA), and non-neovascular exudative fluid. Recent imaging modalities—including optical coherence tomography (OCT), fundus autofluorescence (FAF), and OCT angiography (OCTA)—have uncovered features with important prognostic implications that are currently misclassified with respect to AMD. For example, eyes with RPD or AVL in the absence of drusen are currently misclassified as “early AMD” or excluded altogether, despite their high risk of progression. Similarly, the ambiguous status of non-exudative MNV, which carries both protective and harmful potential, highlights the need for greater granularity. The Classification of Atrophy Meetings (CAM) group has also introduced refined OCT-based definitions such as iRORA and cRORA, underscoring early degenerative changes that precede geographic atrophy. Moreover, novel entities like non-neovascular intraretinal or subretinal fluid challenge the assumption that exudation is synonymous with neovascular AMD. This review synthesizes recent evidence highlighting the limitations of current classification systems in light of these advances. Furthermore, we emphasize that intermediate AMD, currently treated as a uniform category, actually encompasses highly heterogeneous phenotypes with distinct risks and trajectories. A more nuanced, imaging-integrated classification system is urgently needed to improve disease staging, identify high-risk eyes, and ensure appropriate patient selection for emerging therapies. Such a framework would not only better reflect the complex natural history of AMD but also facilitate the regulatory shift toward continuous, quantitative endpoints in clinical trials.

**Supplementary Information:**

The online version contains supplementary material available at https://doi.org/10.1007/s00417-026-07335-9.

## Key messages

*****What is known***:**

- The classification of AMD is crucial, as both prognosis, visual outcomes, and treatment strategies depend on disease stage.

*****What is new***:**

- Advances in multimodal imaging have greatly enhanced our understanding of AMD, leading to the identification of several novel disease features.
- The broadening spectrum of AMD-associated lesions has introduced challenges in fitting various clinical scenarios into the current classification system.

**Supplementary Information:**

The online version contains supplementary material available at https://doi.org/10.1007/s00417-026-07335-9.

## Introduction

Age-related macular degeneration (AMD) is the leading cause of irreversible vision loss in people aged older than 55 in developed countries, with a worldwide prevalence expected to reach 288 million by 2040 [1–3]. AMD progresses through sequential clinical stages that have historically been described using standard color fundus photograph (CFP), which served as the basis for several classification systems developed over the years [3–7].

Among these, the clinical classification proposed in 2013 by the Beckman Initiative for Macular Research Classification Committee represents a widely adopted consensus system. Designed for broad clinical applicability, the Beckman classification relies solely on findings from standard CFP or clinical examination to stratify patients into five stages, based on the presence of certain AMD-related lesions within two-disc diameters from the fovea (Supplemental Table 1). According to its criteria, early AMD is defined by the presence of medium-sized drusen (63–125 μm), while intermediate AMD includes eyes with large drusen (> 125 μm) with or without pigmentary changes, or medium-sized drusen accompanied by pigmentary changes. Advanced, or late, AMD is marked by the development of geographic atrophy (GA) or macular neovascularization (MNV). Currently, geographic atrophy (GA) is defined by atrophy of the outer retina and retinal pigment epithelium (RPE), whereas so called neovascular AMD in late-stage disease is characterized by signs of exudation. In this review, we will also discuss additional entities. As stated by the authors [2], some pathological changes commonly present in AMD eyes, such as reticular pseudodrusen (RPDs), also known as subretinal drusenoid deposits (SDDs) on structural OCT, and acquired vitelliform lesions (AVLs) have not been included in the classification.

Classifying AMD into well-defined stages holds critical importance for both clinical practice and research. Accurate staging enables the application of evidence-based therapeutic strategies tailored to disease severity. The clinical course of AMD is highly heterogeneous, with some eyes remaining stable for years while others progress rapidly to advanced stages. The precise classification of eyes into intermediate AMD is critical to ensure accurate patient selection and to optimize future clinical trials aiming at studying preventive treatments for intermediate AMD.

An essential limitation of the current Beckman classification is its inability to account for certain AMD phenotypes that do not present with drusen. For instance, some eyes exhibit only RPD without conventional drusen, others may show RPD in combination with AVLs and in the absence of drusen. Despite the absence of traditional hallmark features, these eyes are unequivocally affected by AMD and have been shown to carry a high risk of progression to late AMD stages. Under the Beckman criteria, however, such eyes where classical drusen are absent can only be classified as intermediate AMD if pigment abnormalities are present; otherwise, they are relegated to “early AMD” or excluded from the classification entirely. This represents a significant shortcoming, leading to the under-recognition of high-risk phenotypes that merit closer monitoring and potentially earlier intervention. In parallel, the current classification system also groups under the intermediate AMD category several entities with likely distinct pathophysiology and clinical trajectories, such as eyes with non-exudative MNVs, those with ambiguous forms like exudative but non-neovascular AMD, or eyes with basal laminar deposits (BLamD), a known risk factor for progression to GA. Such cases may not follow the same clinical progression history as non-exudative non-neovascular intermediate AMD and could require tailored management approaches. Intermediate AMD therefore encompasses multiple entities, each likely characterized by distinct pathophysiology and different clinical trajectories.

Emerging therapeutic trials in intermediate AMD are increasingly guided by regulatory requirements that favor continuous, quantitative outcomes over event-based endpoints. The U.S. Food and Drug Administration (FDA) has emphasized the need for endpoints that capture gradual disease progression, such as changes in the slope of lesion growth, rather than binary events like conversion to late AMD. In 2025, the Ryan Initiative for Macular Research (RIMR) proposed consensus guidelines for next-generation intermediate AMD trials, suggesting *en face* OCT as an appropriate imaging modality to follow these patients, with key endpoints including choroidal hypertransmission defects (hyperTDs) and ellipsoid zone (EZ) integrity [3, 4]. Since these biomarkers are only detectable with OCT, both patient selection and endpoint assessment should be conducted using the same modality.

Several continuous quantitative imaging biomarkers have already demonstrated structure–function correlations and, in some cases, regulatory acceptance in recent AMD trials. OCT-derived metrics such as ellipsoid zone (EZ) loss rate, and hypertransmission defects (hyperTDs) development/expansion consistently correlate with declines in visual sensitivity and microperimetry performance, while offering better interobserver variability than qualitative assessments. Their value is supported by their use as primary endpoints in major clinical trials evaluating patients with early/intermediate AMD and GA [5–19].

Additionally, the size of drusen measured on CFP often does not correspond to that measured on OCT, whether in terms of apical height or basal width [20, 21]. Therefore, including new imaging techniques—such as OCT, OCTA, fundus autofluorescence (FAF), and others—in AMD classification result paramount.

Beyond its lack of integration with modern imaging, the Beckman classification treats all intermediate AMD eyes as a single group. Yet, strong evidence shows that intermediate AMD includes diverse phenotypes with significantly different risks of progression. Several biomarkers, such as drusen volume, the area of hyperreflective foci (HRF), RPDs, drusenoid PEDs, ghost drusen and AVLs, are all associated with increased risk of advancing to geographic atrophy or neovascular AMD [22]. By ignoring these features, the Beckman system lacks the granularity needed to stratify progression risk, an essential step for both clinical management and research.

The heterogeneous clinical trajectories observed in intermediate AMD are quantitatively supported by multiple longitudinal cohort studies. As displayed in Table 1, different phenotypes show markedly different risks of progression to late AMD, even when classified within the same intermediate stage by current AMD classification systems. Table 1 summarizes relative risk estimates reported in large observational studies, including hazard ratios ranging from two-fold (e.g., RPD, AVL and HRF) to more than six-fold (e.g., iRORA), as well as annualized progression rates [23]. These data underscore that intermediate AMD is not a single clinical entity but a collection of distinct phenotypic pathways with variable prognostic implications.

**Table 1:** Progression risk to late AMD according to different biomarkers

Phenotype	Hazard ratio	Annualized risk of late AMD
RPD/SDD	2.15	6–7%
AVL	2.61	8–9%
HRF	5.21	16.5%
iRORA	12.1	46%

Taken together, these observations reveal substantial gaps in the current AMD classification systems. The aim of this review is to highlight a number of limitations in light of recent advances in imaging and disease understanding. By expanding on the scenarios outlined above, we will examine underrecognized and borderline phenotypes that challenge the existing staging framework.

The growing emphasis on precision in AMD research highlights the need for automated, quantitative, and reproducible imaging metrics. Automated OCT-based analyses mitigate interobserver variability and enable continuous, high-repeatability measurements. Recent work introducing fully quantitative OCT phenotyping frameworks demonstrates high test–retest reliability and cross-dataset validation, positioning these biomarkers as strong candidates for future regulatory-grade endpoints [24–26]. As FDA guidance increasingly favors continuous quantitative measures, such automated pipelines are likely to become central to early-intervention trial design, enabling more sensitive detection of treatment effects and scalable phenotyping across centers.

## Key considerations beyond AMD classification

Additionally, accurate differentiation between AMD and mimicking entities—such as pachychoroid spectrum diseases or inherited macular disorders presenting with drusen-like or vitelliform features—is essential to prevent misclassification. Incorrectly categorizing these conditions as AMD may lead to inappropriate management and risks enrolling non-AMD patients in clinical trials, thereby confounding trial outcomes.

Moreover, before expanding this review to discuss important phenotypes that are currently not included in AMD classification systems, we would like to emphasize the following points regarding (i) artefacts and (ii) co-morbidities, in order to clearly distinguish them from the AMD entities presented.

Multimodal imaging, while essential for comprehensive AMD characterization, also presents important limitations that should be acknowledged. Ultra-widefield SS-OCTA, for instance, has been shown to exhibit previously unrecognized linear shadowing artifacts caused by patient eyelashes, producing false-positive regions of apparent choriocapillaris hypoperfusion on en face imaging and potentially leading to erroneous perfusion assessments [27]. Such modality-specific artifacts highlight how technical factors can affect the reliability of quantitative and qualitative interpretation in AMD research.

These challenges become even more significant in the presence of comorbid retinal diseases. Recent evidence demonstrates that diabetic retinopathy alters choriocapillaris perfusion patterns in eyes with type 1 MNV secondary to AMD, with diabetic eyes showing greater baseline hypoperfusion and an absence of reperfusion after anti-VEGF loading compared with nondiabetic eyes. Specifically, while nondiabetic AMD eyes exhibited a significant reduction in flow-deficit percentage in the choriocapillaris ring surrounding the dark halo following aflibercept therapy, diabetic eyes showed no meaningful change, indicating impaired vascular recovery. These findings suggest that DR not only modifies multimodal imaging signatures—particularly in OCTA-based perfusion metrics—but may also affect disease progression and therapeutic responsiveness [28]. Recognizing both technical artifacts and biological confounders) is essential for accurate interpretation of multimodal imaging, especially in heterogeneous populations and in multicenter studies where standardized acquisition and artifact-aware quality control are critical.

## Reticular pseudodrusen

Reticular pseudodrusen (RPD) describe the clinical fundus appearance of lesions that correspond to subretinal drusenoid deposits (SDD) on OCT. In this review, we use the term “RPD,” acknowledging that it reflects the clinical presentation of SDD observed on OCT. The first description of RPD dates back to 1990, when it was hypothesized that these lesions represented a choroidal reflex observed during fundoscopic examination [29]. Over the years, various terms have been used to describe these lesions, including reticular drusen, reticular pseudodrusen, and reticular macular disease [30]. Nearly two decades after the initial observation, RPD were identified on OCT as hyperreflective lesions located between the retinal pigment epithelium (RPE) and the neuroretina and for this also named subretinal drusenoid deposits [31].

Although RPDs may be observed in other conditions, they are a hallmark finding in AMD, independent of drusen presence. Typically bilateral, these lesions predominantly affect the perifoveal region and retinal periphery, often leading to nyctalopia in affected individuals [32]. Major risk factors for RPD include genetic predisposition, increasing age, and choroidal hypoperfusion [32].

Importantly, RPDs represent a recognized risk factor for the progression to late AMD [33]. Notably, this risk persists even in the absence of conventional drusen [34]. In the latter study, the authors monitored macular changes over time in eyes exhibiting RPD without any drusen and found that these eyes carry a substantial risk of progressing to late AMD, including GA or MNV, particularly type 2 or type 3 MNV [34].

RPD demonstrate a distinctive spatial distribution, preferentially affecting the superior and superior-temporal macular regions, where they frequently form organized reticular configurations. This pattern offers diagnostic utility and may be attributable to underlying choroidal perfusion gradients.

Indeed, emerging evidence indicates that early choriocapillaris flow impairment—visible on OCTA as flow deficits—may precede the appearance of RPD [35]. Integrating these vascular perturbations within an imaging-based diagnostic framework may allow for the identification of high-risk eyes before clinically appreciable structural alterations arise.

**Fig. 1:**
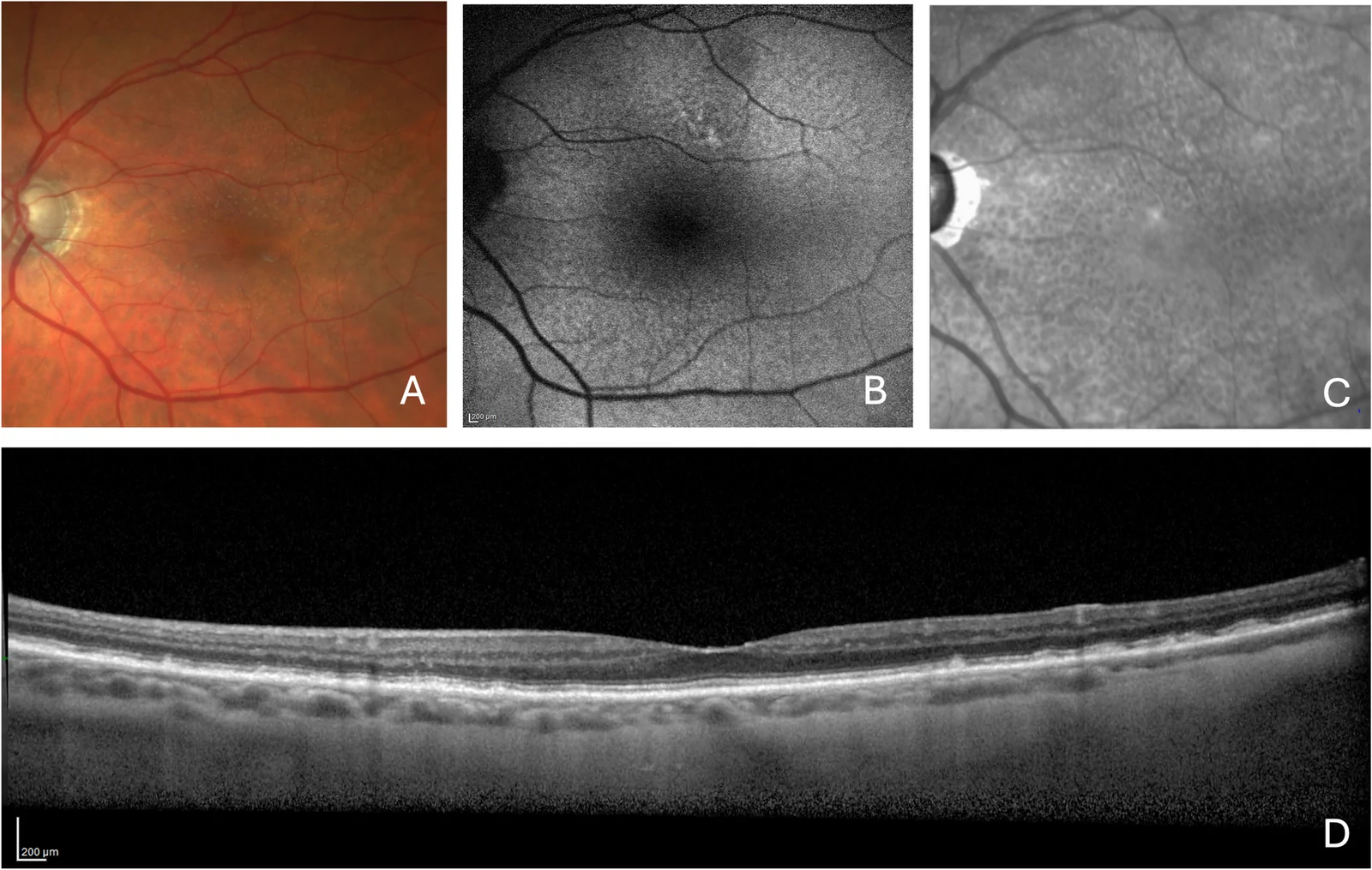
Multimodal imaging of the left eye in a patient with reticular pseudodrusen and no evidence of drusen. Color fundus photography (Clarus 700, Carl Zeiss Meditec) (**A**) shows subtle hypopigmented macular spots, which are more clearly visible on BAF (Heidelberg Engineering) (**B**) and NIR (Heidelberg Engineering) (**C**). The OCT B-scan through the fovea (Heidelberg Engineering) (**D**) confirms the presence of subretinal drusenoid deposits

The lack of drusen in the presence of RPDs in AMD poses challenges in classifying such eyes within current AMD classification systems, which primarily rely on the presence of drusen for staging (Fig. 1). Assuming that early AMD is characterized by the presence of medium-sized drusen (≥ 63 and < 125 μm) without pigmentary abnormalities, and that individuals with large drusen or pigmentary abnormalities associated with at least medium-sized drusen are classified as having intermediate AMD, this clinical scenario does not fit into either of these categories.

## Acquired vitelliform lesions in the absence of drusen

Vitelliform material can accumulate in the context of inherited retinal diseases—most notably in Best macular dystrophy [36]—but it is also observed in various acquired retinal conditions, including AMD, tractional maculopathies, and central serous chorioretinopathy [37]. In these acquired settings, the deposits are referred to as pseudovitelliform or acquired vitelliform lesions [37].

Clinically, AVLs present as yellowish, slightly elevated lesions beneath the fovea on fundus examination and as hyperautofluorescent on FAF. On structural OCT, they appear as dome-shaped subretinal hyperreflective material (SHRM), often accompanied by irregularities of the RPE [38]. AVLs are relatively common in patients with early or intermediate AMD, with a reported prevalence of approximately 4–5% in a recent study [39], and are typically associated with the presence of RPD [40, 41].

**Fig. 2:**
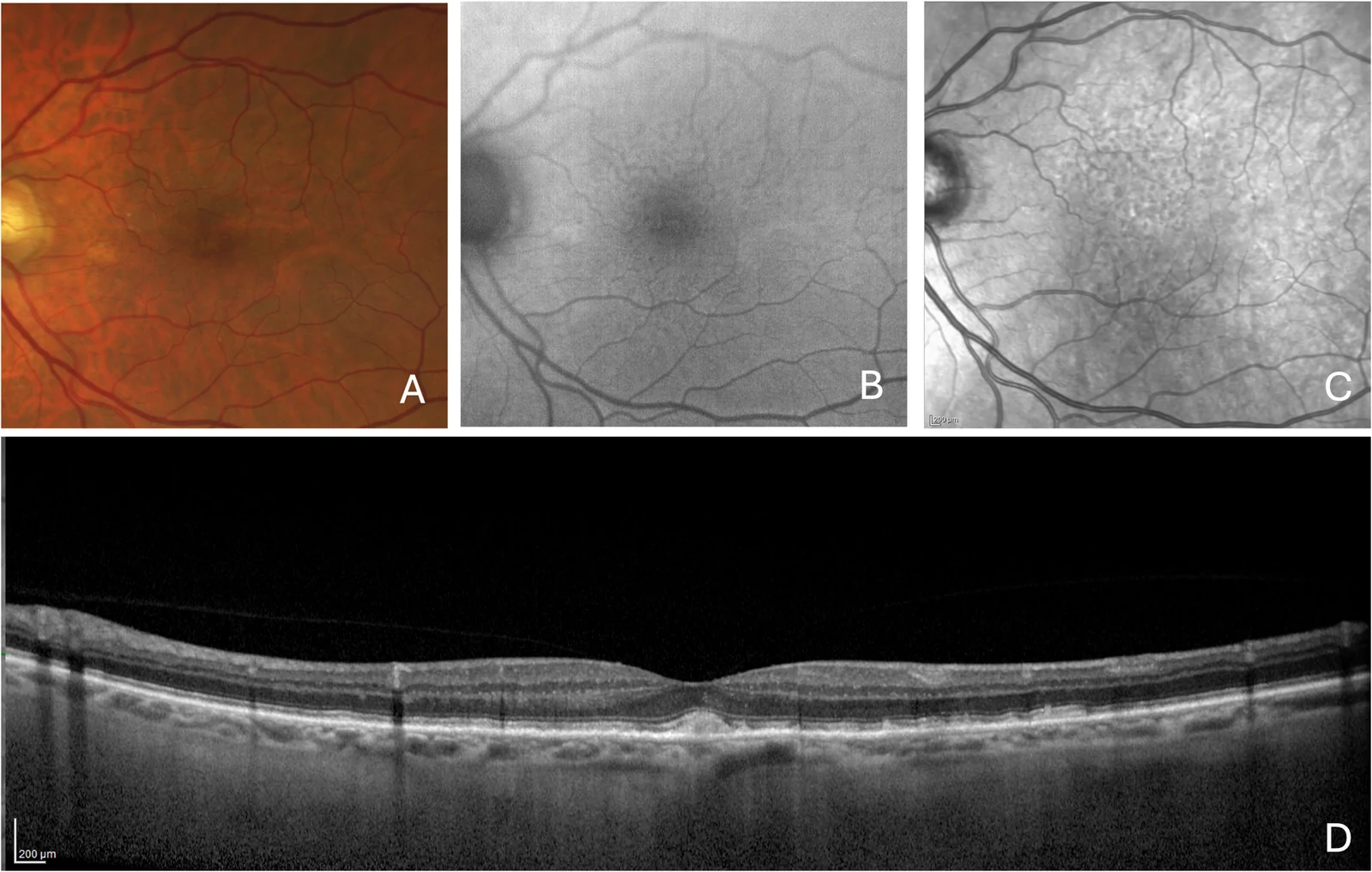
Multimodal imaging of the right eye in a patient with an acquired vitelliform lesion. CFP (Clarus 700, Carl Zeiss Meditec) (**A**) reveals a yellowish lesion beneath the fovea and fine hypopigmented spots, i.e. pseudodrusen, superiorly to the fovea. The lesion appears mildly hyperautofluorescent on GAF image (Clarus 700, Carl Zeiss Meditec) (**B**). On Near infrared image (Heidelberg Engineering) (**C**) the lesion appears hyperreflective while pseudodrusen appear hyporeflective. The structural OCT (Heidelberg Engineering) (**D**) shows a dome-shaped subretinal hyperreflective material, accompanied by irregularities of the retinal pigment epithelium (RPE), with some SDD in the perifoveal region

Eyes with intermediate AMD and AVL tend to show a higher prevalence of pachyvessels and increased choroidal thickness compared to those without AVLs [41]. Interestingly, it is not uncommon to encounter cases presenting with both RPD and AVL in the absence of drusen (Fig. 2).

Patients with AVLs are at increased risk of progressing to late-stage AMD. Specifically, AVLs can enlarge over time and eventually undergo resorption, sometimes leaving void spaces with no MNV. The presence of RPD and increased AVLs’ height has been shown to significantly elevate the risk of atrophy developing at the lesion site following resorption [40]. Importantly, atrophic lesions that emerge following AVL collapse often exhibit distinct growth dynamics compared to GA not associated with AVLs, with potentially slower centrifugal expansion. Spaide et al. [34] analyzed the progression of eyes with subretinal drusenoid deposits (SDD – the OCT finding in presence of RPD) in the absence of conventional drusen and found that a significant proportion developed subfoveal AVLs as the disease advanced. During the resorption phase, a hyperpigmented area often formed, which was associated with a higher risk of progression to type 3 macular neovascularization. These findings highlight the prognostic significance of AVLs in the progression of AMD.

As previously noted for eyes with RPD but no drusen, the presence of AVLs in the absence of drusen similarly complicates the classification of these eyes within existing AMD staging systems, which predominantly depend on the presence of drusen for categorization.

## Non-exudative macular neovascularization

To avoid terminological ambiguity, it is essential to clearly differentiate among conceptually distinct entities. Non-exudative MNV refers to angiographically or OCTA–positive neovascular complexes that do not exhibit exudation on structural OCT [42]. In contrast, non-neovascular exudation describes the presence of intraretinal or subretinal fluid on OCT in the absence of any detectable MNV on OCTA, FA, or ICGA. These definitions are applied consistently throughout the manuscript.

**Fig. 3:**
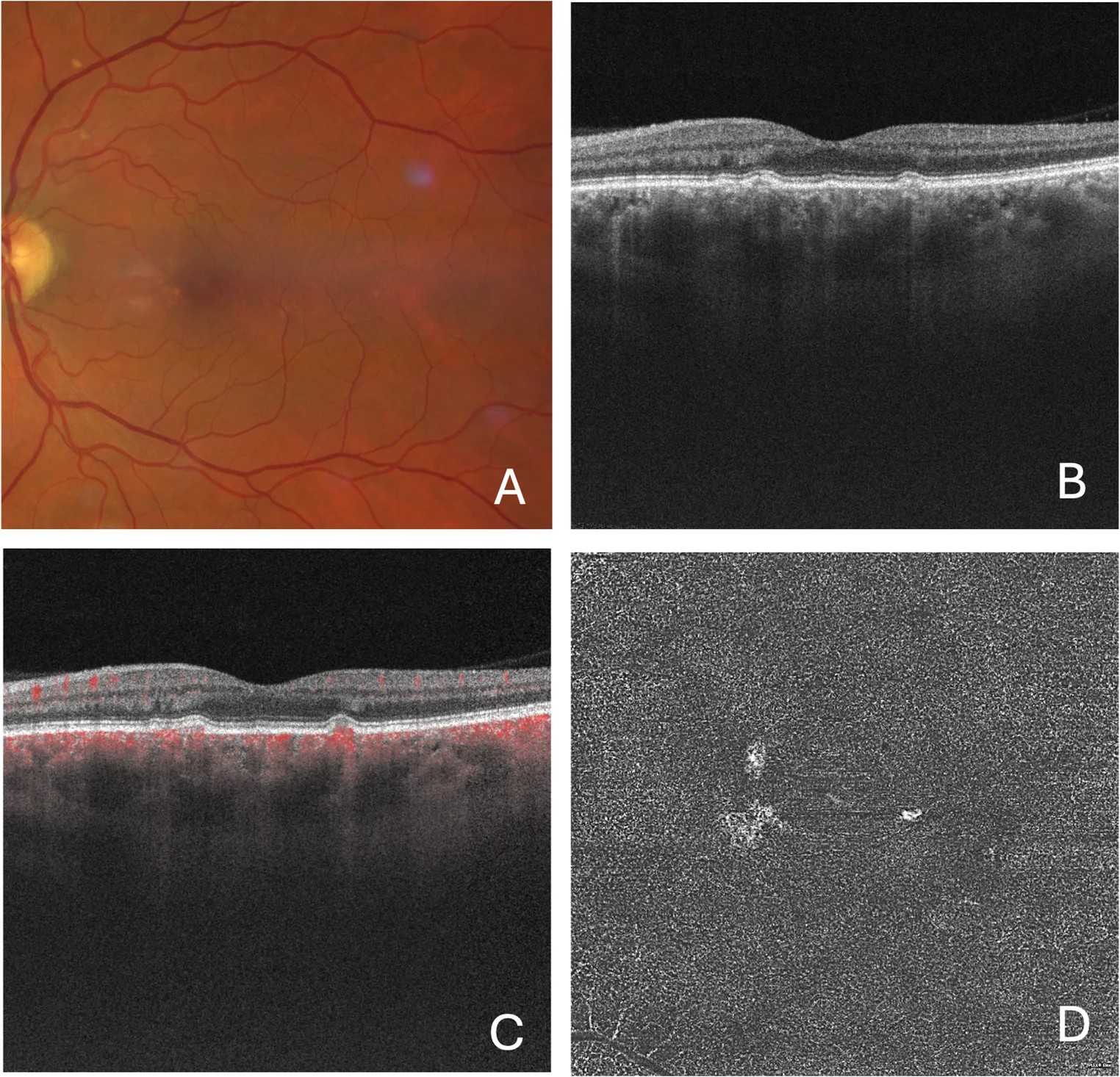
Multimodal imaging of the left eye in a patient with neovascular, non-exudative age-related macular degeneration (AMD). Color fundus photography (Clarus 700, Carl Zeiss Meditec) (**A**) reveals hypopigmented lesions resembling drusen. Structural OCT (Plex Elite 9000, Carl Zeiss Meditec) (**B**) identifies two small pigment epithelial detachments (PEDs). OCT angiography (OCTA) (Plex Elite 9000, Carl Zeiss Meditec) demonstrates blood flow within the PEDs, visible on both the B-scan (**C**) and the *en face* RPE–RPE fit slab (Plex Elite 9000, Carl Zeiss Meditec) (**D**), without any OCT evidence of exudation

Current classifications, e.g., Beckmann classification [2], define neovascular AMD as a late-stage form of the disease, characterized by the presence of MNV, without explicitly stating whether exudation is required for this definition. This ambiguity raises uncertainty about whether non-exudative MNV should be classified as intermediate or late AMD. Figure 3 shows a case with non-exudative MNV in AMD. Non-exudative MNVs in AMD were first identified in postmortem eyes in the 1970s [43, 44]. However, it was not until the introduction of advanced imaging modalities that non-exudative MNV was thoroughly studied and characterized. The prevalence of non-exudative MNV has been reported to be as high as 27% in patients with AMD and exudative MNV in the fellow eye [45].

In the presence of non-exudative MNV, the BCVA is generally unaffected [46]. On fundoscopic examination patients frequently present with drusen and pigmentary abnormalities [47]. On OCT, non-exudative MNV lesions appear with a shallow RPE elevation with heterogeneous hyperreflective material in between the RPE and Bruch’s membrane. This OCT feature is referred to as thick double layer sign (DLS), whose specificity and sensitivity can reach 88% to determine the presence of a type 1 non-exudative MNV [45], in contrast with the thin DLS which carries a higher risk of progression to cRORA. The sensitivity and specificities are even superior with OCTA, more efficacious than OCT in identifying these lesions [48]. Of note, also type 3 MNV can also be diagnosed in the absence of clear OCT signs of exudation (i.e., this entity referred as non-exudative type 3 MNV or nascent type 3 MNV) [49, 50], particularly IRF, which typically appears early in this context. Suspicion arises when structural OCT reveals hyperreflective material in the outer retina without associated IRF.

Eyes with non-exudative MNV carry a considerable risk of developing exudation, with a cumulative incidence of 24% at one year, rising to 34.5% at two years [48]. Therefore, while intravitreal anti-VEGF therapy is not recommended in the absence of exudation, close monitoring is strongly advised [46, 48].Conversely, non-exudative type 1 MNV may have a protective role against the progression of GA, potentially by offering metabolic support to the RPE [51].

The prognostic implications of non-exudative MNV depend strongly on lesion type, anatomical location, and perfusion characteristics. Type 1 neovascular complexes, when stable and well-perfused, may provide metabolic support to the outer retina and have been associated with slower GA expansion. In contrast, non-exudative type 3 lesions—often characterized by focal outer retinal hyperreflectivity, minimal flow, and deep capillary plexus congestion—are associated with a substantially higher risk of exudation and progression [52]. The heterogeneity across MNV phenotypes highlights the importance of contextual interpretation rather than a uniform prognostic label.

## Incomplete RPE and outer retina atrophy

The term GA has traditionally referred to late-stage non-neovascular AMD and is defined as an area of hypopigmentation with increased visibility of underlying choroidal vessels on CFP, though lesion size criteria may vary across studies [53, 54]. Due to the challenges in accurately delineating GA borders on CFP, more advanced imaging modalities have been adopted for improved lesion characterization [55]. FAF, in particular, provides clear demarcation between atrophic and non-atrophic areas—atrophic regions appearing hypoautofluorescent—and is now widely used for the detection, monitoring, and quantification of GA [56]. In addition, FAF enables the detection of surrounding changes, such as patterns of hyper- and hypoautofluorescence, that have been shown to correlate with GA lesion growth and offer insight into micro-progression pathways from intermediate AMD to cRORA formation [57]. These peripheral alterations are not readily detectable with other imaging modalities, underscoring FAF’s unique value in prognostication and disease monitoring.

The widespread use of OCT in clinical practice has significantly enhanced our understanding of atrophic processes in AMD. The Classification of Atrophy Meetings (CAM) program proposed four terms to describe AMD-related atrophy: complete RPE and outer retinal atrophy (cRORA), incomplete RPE and outer retinal atrophy (iRORA), complete outer retinal atrophy (cORA), and incomplete outer retinal atrophy (iORA) [55]. This classification is primarily based on OCT features, where the presence of signal hypertransmission, disruption of the RPE, and degeneration of the overlying photoreceptors define areas of RPE and outer retinal atrophy (RORA). A lesion is classified as cRORA when all three features are present in a region measuring at least 250 micrometers in diameter; lesions not meeting the above criteria for cut-off are classified as iRORA [55, 58].

The Beckman classification uses the term GA to describe the late-stage non-neovascular form of AMD (Supplemental Table 1). To align this widely used framework with the CAM definitions, the CAM group proposed using the term GA specifically for areas of cRORA in the absence of neovascularization [55].

**Fig. 4:**
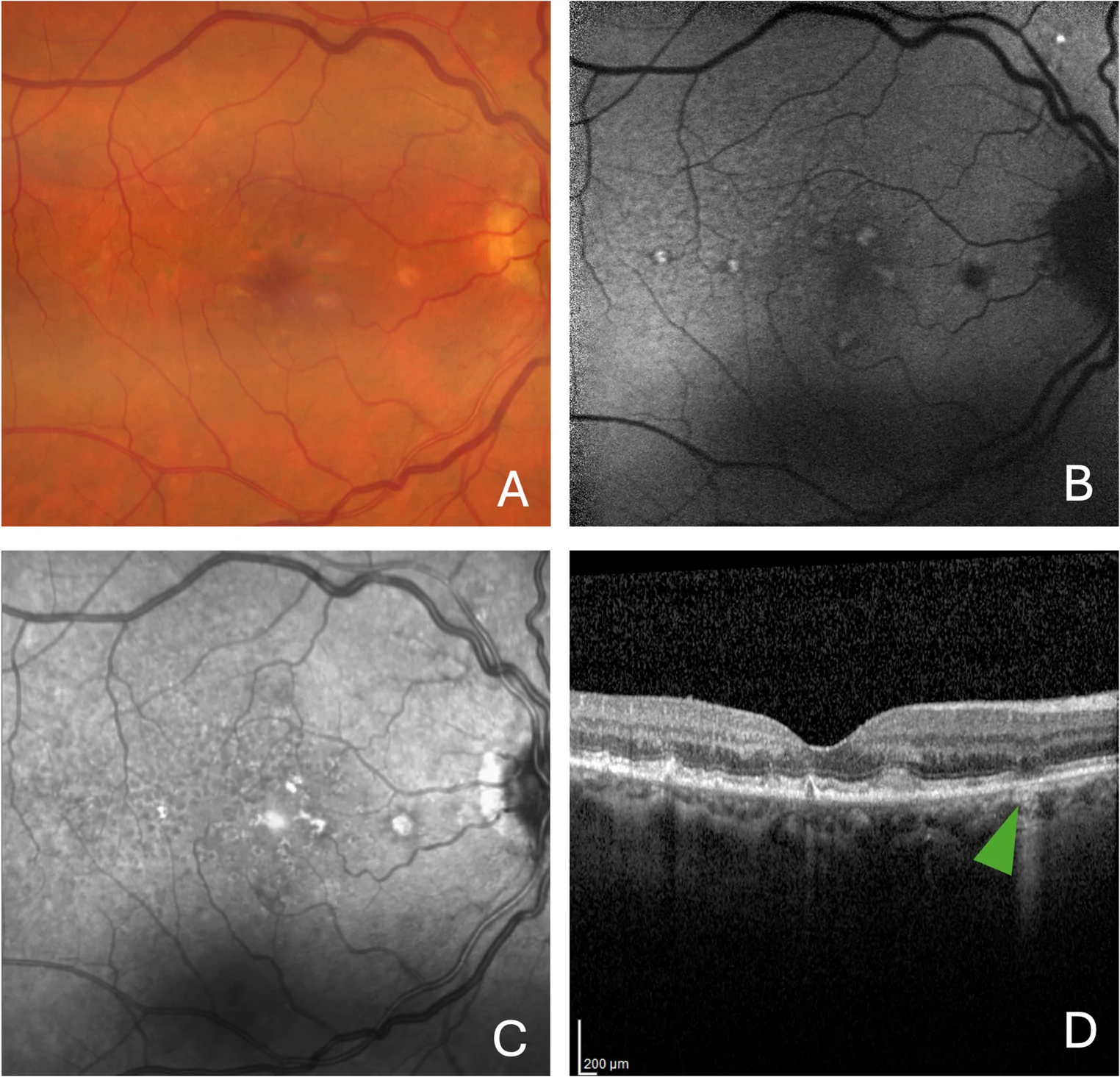
Multimodal imaging of the right eye in a patient with iRORA. CFP (Clarus 700, Carl Zeiss Meditec) (**A**) shows a small hypopigmented area of atrophy nasal to the fovea, corresponding to a region of hypoautofluorescence on BAF (Heidelberg Engineering) (**B**), which appears hyperreflective on the NIR image (Heidelberg Engineering) (**C**). Structural OCT (Plex Elite 9000, Carl Zeiss Meditec) (**D**) confirms the presence of an iRORA lesion (green arrowhead), characterized by loss of the RPE and photoreceptor layer over a width of less than 250 microns, along with a hypertransmission defect

Although the presence of iRORA, which can present with different phenotypic patters, as can be demonstrated by FAF, typically classifies patients as having intermediate AMD (Fig. 4), it is important to note that most incident iRORA lesions progress to cRORA within 24 months, indicating the onset of an irreversible degenerative process [59]. Moreover, areas of iRORA are associated with reduced retinal sensitivity, as demonstrated by microperimetry testing, suggesting that significant functional impairment is already present within these regions [60].

## Exudative intraretinal fluid in early/intermediate AMD or GA with no MNV

Exudative fluid has traditionally been associated with a neovascular origin. However, entities of non-neovascular exudative IRF have been recently observed and described.

In the absence of MNV, non-neovascular exudation is defined by the presence of newly detected intraretinal cystic spaces on structural OCT, accompanied by increased retinal thickness and displacement of adjacent retinal structures [61].

Non-neovascular exudation tends to occur in an older population exhibiting classic features of AMD. In the latter setting, a chronic ischemic state in the retina may develop in areas with significant disruption of the Bruch’s membrane–RPE complex, likely due to functional insufficiency of the choriocapillaris [61, 62]. This insufficiency can elevate intraretinal VEGF levels that may eventually promote leakage from the DVC [61, 62]. This type of exudation may occur with or without associated microvascular anomalies, such as the recently described “deep retinal age-related microvascular anomalies” (DRAMA) [61, 63]. DRAMAs typically present as small-diameter perifoveal capillary dilations with hyperreflective walls located in the inner nuclear layer, or as multiple vascular outpouchings extending posteriorly into the Henle fiber layer, with reflectivity similar to surrounding normal retinal capillaries [63].

**Fig. 5:**
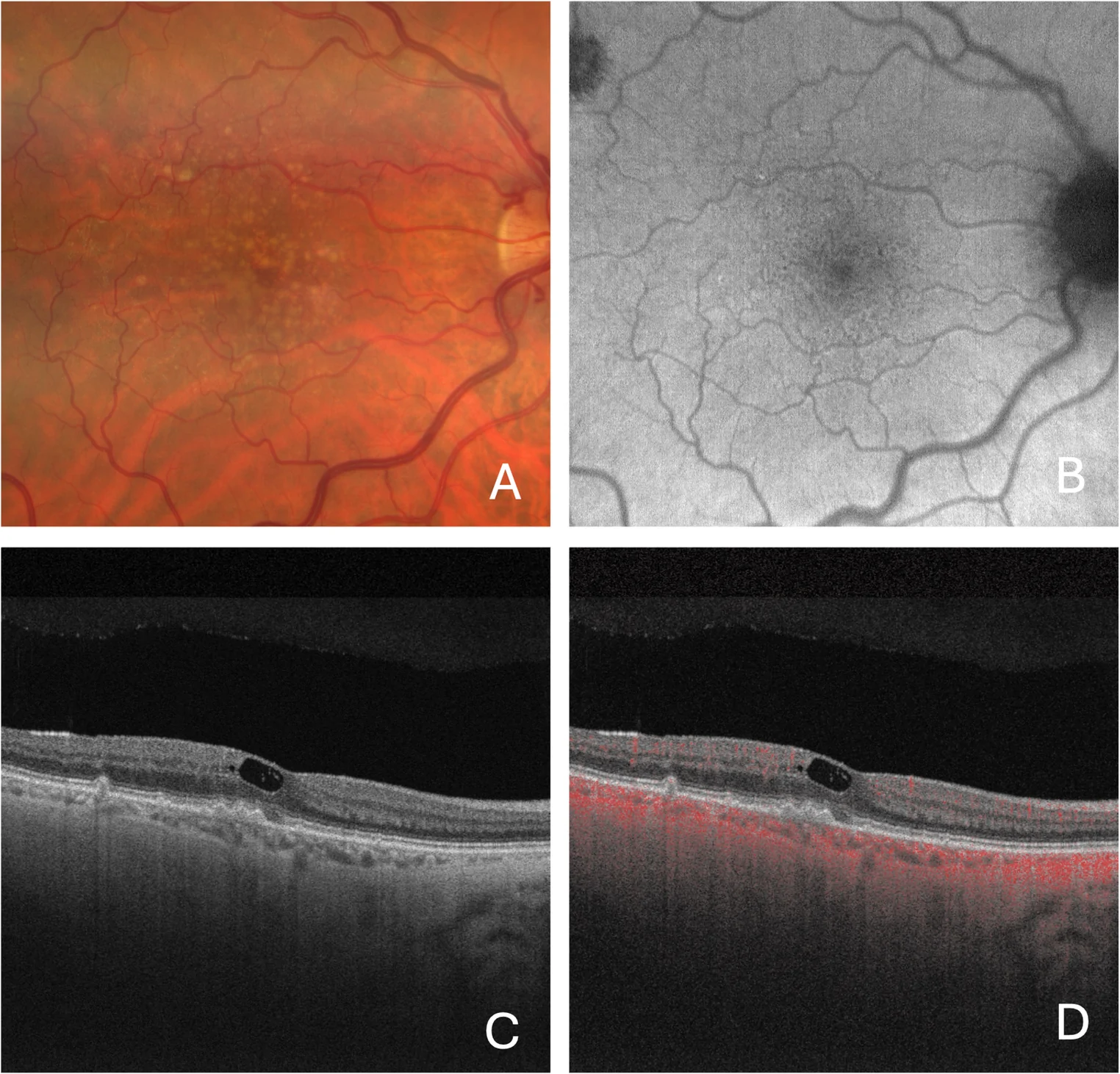
Multimodal imaging of the right eye in a patient with exudative intraretinal fluid in early/intermediate AMD. CFP (Clarus 700, Carl Zeiss Meditec) (**A**) shows large drusen in the macular region, corresponding to hypoautofluorescent areas on the GAF image (Clarus 700, Carl Zeiss Meditec) (**B**). Structural OCT (Plex Elite 9000, Carl Zeiss Meditec) (**C**) reveals the presence of intraretinal fluid (IRF) and drusen. Structural OCTA (Plex Elite 9000, Carl Zeiss Meditec) (**D**) shows no evidence of associated neovascularization

These lesions are not included in the current classification and may therefore appear in early/intermediate AMD or GA, depending on the other features described above (Fig. 5). However, their presence might indicate distinct underlying processes that could be considered in future classification systems.

## Non-exudative subretinal fluid in intermediate AMD or GA

Non-exudative SRF was first described by Hilely and colleagues [64]. This phenomenon is relatively common and is usually associated with large drusenoid PEDs, where the SRF tends to accumulate at the apex or along the margins of the PED. It has been suggested that SRF may arise due to increased separation between the RPE and the CC, leading to RPE hypoperfusion and subsequent dysfunction [64].

**Fig. 6:**
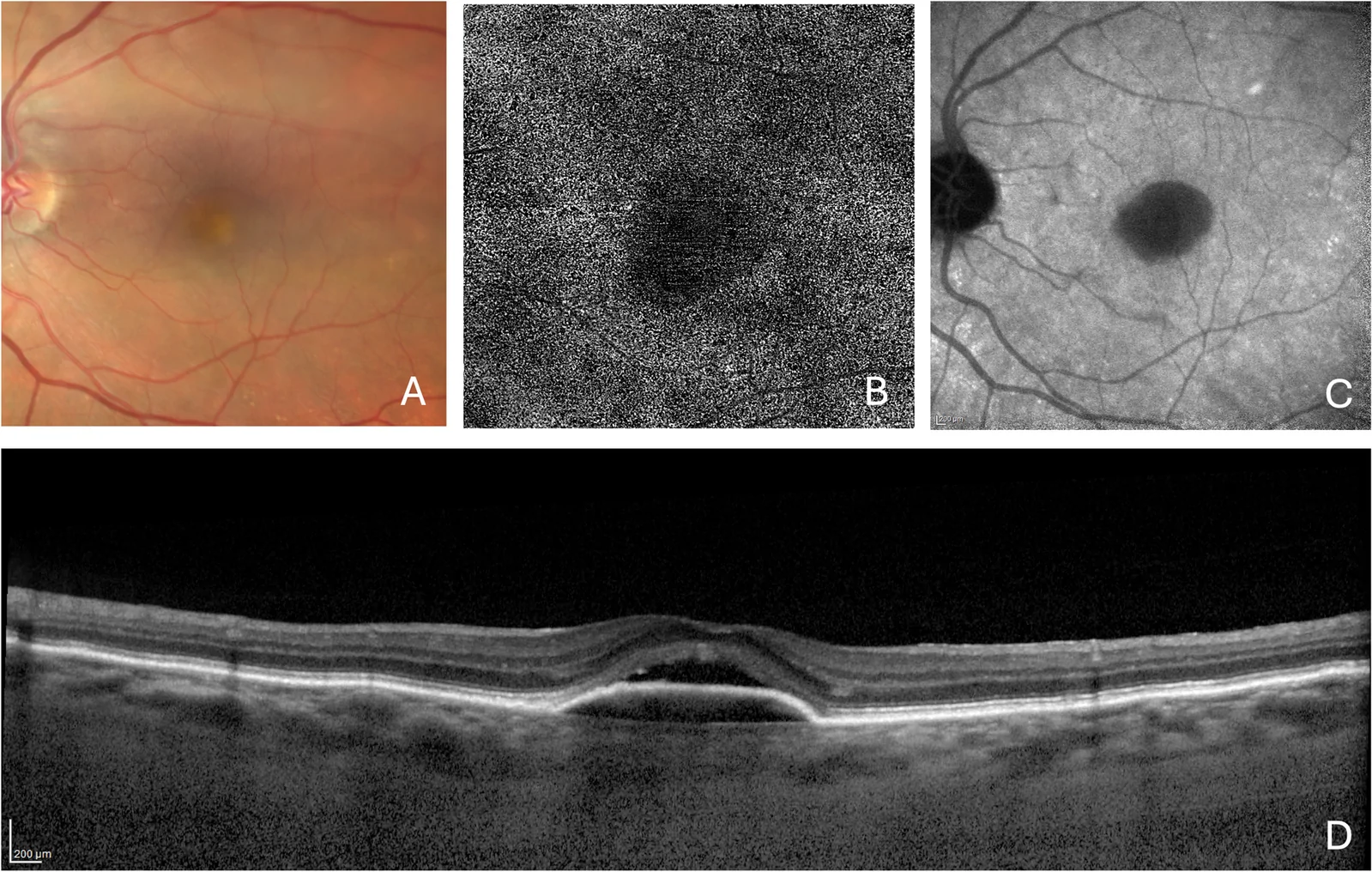
Multimodal imaging of the left eye in a patient with non-exudative subretinal fluid in intermediate AMD. Color fundus photography (Clarus 700, Carl Zeiss Meditec) (**A**) reveals pigmentary changes in the macular region. Neither the en face OCTA image of the ORCC slab (Plex Elite 9000, Carl Zeiss Meditec) (**B**) nor the late-phase ICGA (Heidelberg Engineering) (**C**) shows signs of macular neovascularization. However, structural OCT (Plex Elite 9000, Carl Zeiss Meditec) (**D**) demonstrates the presence of subretinal fluid associated with a pigment epithelial detachment

Regarding cases of exudative intraretinal fluid without evidence of MNV, as mentioned in the previous paragraph, present lesions not currently included in the existing classification (please see example in Fig. 6). They may be observed in early or intermediate AMD, or GA, depending on other associated features. Nevertheless, their presence could reflect distinct underlying mechanisms that could be considered in future classification updates.

## Conclusions

AMD is a common and vision-threatening condition characterized by a variety of lesions that can coexist, resulting in a complex disease process. This complexity has been further heightened by advances in multimodal imaging, which have improved the definition of previously known lesions and led to the discovery of new findings.

The feasibility of integrating additional biomarkers into AMD staging also depends on reproducibility across imaging platforms and intergrader reliability. Several OCT- and OCTA-based metrics (e.g., HRF quantification, choriocapillaris flow deficits, RORA criteria) now show promising reproducibility in multicenter settings, supporting potential regulatory adoption of imaging-based endpoints.

The classification of AMD holds significant importance for both clinical practice and research trials. It is closely linked to the risk of disease progression and visual prognosis, making it a crucial aspect of patient management. However, the increasing complexity of the disease has created challenges in classification, as certain clinical presentations do not clearly fit within existing categories. Therefore, this review highlights the need for a more comprehensive, imaging-integrated classification system that better reflects current clinical realities and research requirements.

## Supplementary Information

Below is the link to the electronic supplementary material.

Supplementary Material 1

## Data Availability

Not applicable.

## Abbreviations and acronyms

AMD: Age related Macular Degeneration
AVLs: Acquired Vitelliform Lesions
BCVA: Best-Corrected Visual Acuity
BLamD: Basal Laminar Deposits
CAM: Classification of Atrophy Meetings
CC: Choriocapillaris
CFP: Color Fundus Photography
cORA: Complete Outer Retinal Atrophy
cRORA: Complete RPE and Outer Retinal Atrophy
DLS: Double Layer Sign
DRAMA: Deep Retinal Age-Related Microvascular Anomaly
DVC: Deep Vascular Complex
EZ: Ellipsoid Zone
FA: Fluorescein Angiography
FAF: Fundus Autofluorescence
GA: Geographic Atrophy
HRF: Hyperreflective Foci
ICGA: Indocyanine Green Angiography
iORA: Incomplete Outer Retinal Atrophy
iRORA: Incomplete RPE and Outer Retinal Atrophy
IRF: Intraretinal Fluid
MNV: Macular Neovascularization
OCT: Optical Coherence Tomography
OCTA: Optical Coherence Tomography Angiography
PED: Pigment Epithelial Detachment
RIMR: Ryan Initiative for Macular Research
RPE: Retinal Pigment Epithelium
RPD: Reticular Pseudodrusen
SDDs: Subretinal Drusenoid Deposits
SHRM: Subretinal Hyperreflective Material
SRF: Subretinal Fluid
VEGF: Vascular Endothelial Growth Factor
