# Supplementary material for: Review of emerging imaging findings to reveal a broader spectrum of lesions in AMD
Source: Graefes Arch Clin Exp Ophthalmol. 2026 Jun 25;264(11):3359–71. doi: 10.1007/s00417-026-07335-9 (PMC13736352; doi:10.1007/s00417-026-07335-9)
Supplement: Supplementary file 1 [file 417_2026_7335_MOESM1_ESM.docx]

**Supplemental table 1. Beckman Classification of AMD**

| **Disease Stage** | **Definition** |
| --- | --- |
| No apparent aging changes | • No drusen • No AMD pigmentary abnormalities* |
| Normal aging changes | • Only small drusen ≤ 63 µm • No AMD pigmentary abnormalities* |
| Early AMD | • Medium drusen > 63 µm and ≤ 125 µm • No AMD pigmentary abnormalities* |
| Intermediate AMD | Large drusen > 125 µm and/or any AMD pigmentary abnormalities* |
| Late AMD | Neovascular AMD and/or GA |

* AMD pigmentary abnormalities: any definite hyper- or hypopigmentary abnormalities associated with medium or large drusen but not associated with known disease entities.
